# Characterization of a novel method for the production of single‐span membrane proteins in *Escherichia coli*

**DOI:** 10.1002/bit.26895

**Published:** 2019-01-19

**Authors:** Sarah M. Smith, Kelly L. Walker, Alexander S. Jones, Corinne J. Smith, Colin Robinson

**Affiliations:** ^1^ School of Life Sciences, University of Warwick Coventry UK; ^2^ School of Biosciences, University of Kent Canterbury UK

**Keywords:** biotechnology, electron microscopy, membrane proteins, protein engineering, twin arginine

## Abstract

The large‐scale production and isolation of recombinant protein is a central element of the biotechnology industry and many of the products have proved extremely beneficial for therapeutic medicine. *Escherichia coli* is the microorganism of choice for the expression of heterologous proteins for therapeutic application, and a range of high‐value proteins have been targeted to the periplasm using the well characterized Sec protein export pathway. More recently, the ability of the second mainstream protein export system, the twin‐arginine translocase, to transport fully‐folded proteins into the periplasm of not only *E*. coli, but also other Gram‐negative bacteria, has captured the interest of the biotechnology industry.

In this study, we have used a novel approach to block the export of a heterologous Tat substrate in the later stages of the export process, and thereby generate a single‐span membrane protein with the soluble domain positioned on the periplasmic side of the inner membrane. Biochemical and immuno‐electron microscopy approaches were used to investigate the export of human growth hormone by the twin‐arginine translocase, and the generation of a single‐span membrane‐embedded variant. This is the first time that a bonafide biotechnologically relevant protein has been exported by this machinery and visualized directly in this manner. The data presented here demonstrate a novel method for the production of single‐span membrane proteins in *E. coli*.

## 1 INTRODUCTION

*Escherichia coli* is responsible for the production of over one‐third of therapeutic proteins (Walsh, 2010). Conventional strategies for the isolation of these heterologous, recombinant proteins from *E. coli* include, expression of soluble proteins in the cytoplasm, expression as insoluble inclusion bodies, or export to the periplasm with subsequent outer membrane rupture to release the periplasmic contents (Pooley, Merchante, & Karamata, 1996). Adoption of the latter approach offers two main advantages: First, the oxidizing environment of the periplasm allows disulfide bonds to form, and second, rupturing of only the outer membrane of Gram‐negative bacteria means there are fewer contaminating cytoplasmic proteins.

The ability of the twin‐arginine translocase (hereafter denoted Tat system) to transport fully‐folded proteins into the periplasm of Gram‐negative bacteria is valued by the biotechnology industry for its potential to simplify the downstream processing of high‐value, biotherapeutic proteins. Initial studies investigated the ability of the Tat translocase to transport a model heterologous protein on a large scale. These studies showed that Tat could export green fluorescent protein (GFP) at levels comparable with conventional methods, and with no large‐scale release of cytoplasmic contents (Matos et al., 2012), confirming this machinery's potential as a viable method for producing heterologous protein.

Most of the Tat system's substrates are soluble proteins, and most studies on the biotechnological exploitation of the system have similarly focused on soluble high‐value protein (Alanen et al., 2015; Browning et al., 2017; Matos et al., 2013). However, the Tat system does have considerable potential for the production of single‐span heterologous membrane proteins, particularly those that contain a globular domain located on the periplasmic face of the inner membrane. Two studies have shown that it is possible to “stall” a Tat substrate at the inner membrane by virtue of a substitution mutation in the signal peptidase cleavage site of a TorA signal peptide (Karlsson et al., 2012; Ren, Patel, & Robinson, 2013). Both studies used biochemical approaches to demonstrate that a Tat substrate remained membrane‐anchored with its mature domain exposed at the periplasmic side of the inner membrane. These data reveal a potential for the Tat translocase to be used in the production of membrane‐bound proteins or for bacterial cell surface display technologies.

Bacterial surface display involves the presentation of a recombinant protein or peptide on the surface of microorganisms, most common of which is *E. coli*, owing to its ease of genetic manipulation and ability to produce recombinant proteins in high yields. As the name suggests, this presentation technology results in the protein of interest being positioned on the surface of the cell where it is available for interaction with any externally added substrate, which does not have to penetrate the membrane. Implementation of this technique has proven beneficial for numerous applications, including biofuel production and protein library screening (Wu, Mulchandani, & Chen, 2008).

A prerequisite of cell surface display in *E. coli* is that the protein of interest is successfully transported across the inner membrane and into the outer membrane; however, the presence of lipopolysaccharides can sterically hinder interactions between expressed protein and binding partner, and assembly at cellular appendages (e.g., flagella or pilli) can disrupt assembly of the recombinant protein (Chen & Georgiou, 2002). Therefore, alternative methods are required that avoid the pitfalls of displaying a protein at the outer membrane. An anchored periplasmic expression is one such alternative that involves the display of heterologous protein in the periplasm of *E. coli*, which is tethered to the inner membrane by a lipoprotein targeting motif (Mazor, Van Blarcom, Iverson, & Georgiou, 2008).

The vast majority of proteins that are presented using this method are exported out of the cytoplasm in an unfolded state via the Sec translocon (Driessen & Nouwen, 2008). However, this export is reliant on the recombinant protein refolding to a native, biologically active conformation in the periplasm. Use of Tat machinery to traverse the inner membrane avoids this issue, since this machinery contains an inherent quality control mechanism that only allows soluble, correctly folded proteins to be transported from the *E. coli* cytoplasm, across the inner membrane, and into the periplasm (DeLisa, Tullman, & Georgiou, 2003; Fisher, Kim, & DeLisa, 2006). Whereas inner membrane display does require outer membrane removal to gain access to the recombinant protein, in instances where cell surface display on the outer membrane of bacteria is impossible, or hugely inefficient, this “extra‐step” of membrane removal far outweighs the difficulty of displaying protein at the outer membrane. Such an approach has previously been used when displaying scFVs via Tat machinery (Karlsson et al., 2012; Moghaddam‐Taaheri, Ikonomova, Gong, Wisniewski, & Karlsson, 2016).

This inherent quality control mechanism of the Tat machinery, in combination with data showing that a signal peptide mutation in a Tat substrate is able to stall a Tat precursor at the inner membrane (Ren et al., 2013), highlights the potential for Tat machinery in cell surface display technology.

To investigate this further, we used biochemical and electron microscopy approaches to analyze the export of a biopharmaceutical, human growth hormone (hGH), which has been shown to be successfully transported by the Tat machinery (Alanen et al., 2015). hGH is a single polypeptide chain of 191 amino acids and is one of the most important hormones in the human body, possessing vital roles in numerous biological processes including cell metabolism and proliferation (Kassem, Blum, Ristelli, Mosekilde, & Eriksen, 1993). Recombinant hGH is used therapeutically to treat hGH deficiency and a range of genetic disorders (Sanchez‐Ortiga, Klibanski, & Tritos, 2012; Spiliotis, 2008; Takeda et al., 2010; Vogt & Emerick, 2015).

Only two heterologous proteins have been shown to be anchored in the cell envelope of *E. coli* via the Tat machinery. Maltose binding protein (MBP) was anchored at the inner membrane via the addition of a 22‐residue C‐terminal transmembrane helix (from the native Tat substrate, HybO; Karlsson et al., 2012). The same study also detected scFv13 (human antibody fragment specific for ß‐galactosidase) at the inner membrane when a FLAG tag was positioned between the signal peptide and the scFv domain.

In this study, we have sought to achieve stable expression of a noncleavable Tat precursor at the inner membrane of *E. coli* with no alterations to the mature protein, and we have used immunogold electron microscopy to precisely localize the target proteins for the first time.

Direct immunogold labeling revealed that overexpressed, membrane‐targeted hGH exhibits a uniform distribution in the inner membrane of *E. coli*. The data confirm that this type of approach can be used for the display of globular protein domains on the periplasmic face of the inner membrane.

## 2 MATERIALS AND METHODS

### 2.1 Export assays of hGH in *E. coli* cells

*E. coli* cells (transformed with a plasmid containing either wild‐type (WT) or mutant precursor hGH (Alanen et al., 2015), were cultured in 1 L Luria Broth (LB) media and induced with 1 mM arabinose (or 1 mM isopropyl β‐D‐1‐thiogalactopyranoside [IPTG]) and cultured for 2 hr. At given intervals (0 hr, 1 hr, 1 hr 15 min, 1 hr 30 min, 1 hr 45 min, and 2 hr), 10 ml of culture was harvested and the cell pellet was normalized against an optical density of 600 nm (e.g., for an optical density of 0.6 at 600 nm, the cell pellet was resuspended in 0.6 ml of disruption buffer [100 mM Tris‐acetate pH 8.2, 500 mM sucrose, and 5 mM ethylenediaminetetraacetic acid {EDTA}]).

### 2.2 Fractionation of *E. coli* cells and detection of hGH protein

*E. coli* cells were fractionated into periplasmic, cytoplasmic, and membrane fractions using the lysozyme/cold osmotic shock method (Randall & Hardy, 1986). The resulting fractions were analyzed by sodium dodecyl sulfate‐polyacrylamide gel electrophoresis (SDS‐PAGE).

Once the periplasmic, cytosolic, membrane, and insoluble samples had been analyzed using SDS‐PAGE, the proteins were transferred from acrylamide gels to polyvinylidene difluoride (PVDF) membranes via semi‐dry western blot analysis apparatus. The membranes were blocked overnight in a solution of 5% (w/v) dried milk in phosphate‐buffered saline (PBS)‐Tween [0.8% NaCl (w/v), 0.02% KCl (w/v), 0.144% Na_2_HPO_4_ (w/v), 0.024% KH_2_PO_4_ (w/v), and 0.2% Tween‐20 (v/v), pH 7.2] and then incubated with primary anti‐hGH antibody (1:20,000, Tris‐buffered saline (TBS)/Tween [0.24% Tris (w/v), 2.5% NaCl (w/v), and 0.2% Tween‐20 (v/v), pH 8.4]) for 1 hr before washing. Membranes were incubated with anti‐rabbit‐horseradish peroxidase (HRP) conjugate (Promega, WI) for 1 hr at room temperature (RT), and subsequently washed. Finally, immunoreactive bands were detected using ECL^TM^ detection reagents according to the manufacturer's instructions. X‐ray films were developed using an AGFA Curix 60 automatic developer as directed by the manufacturer's instructions.

### 2.3 Fractionation of *E. coli* cells and detection of LacI and maltose binding protein

The detection of LacI and maltose binding protein in *E. coli* cells was conducted as described in Section 2.2, with the only difference being the primary antibody used. To detect LacI and maltose binding protein, anti‐LacI and anti‐maltose binding protein primary antibodies were used (both at 1:20,000, TBS/Tween [0.24% Tris (w/v), 2.5% NaCl (w/v), and 0.2% Tween‐20 (v/v), pH 8.4]), respectively.

### 2.4 Preparation of *E. coli* cells for visualization by electron microscopy

Chemical fixation of *E. coli* cells: *E. coli* cells (overexpressing either WT or mutant precursor hGH) were resuspended in aldehyde fixative (0.25% glutaraldehyde/4% formaldehyde) and low‐melting temperature agarose at 1:1 volume ratio. Agarose‐enrobed cells were then harvested and incubated on ice until the agarose had set. Cells were diced into 1 mm^3^ pieces using a clean razor blade and resuspended in fresh aldehyde fixative for overnight fixation at 4°C. Chemically fixed cells were washed for 1–2 hr in 0.15 M sodium cacodylate/HCl buffer pH 7.4 at 4°C to remove aldehyde fixative. The reaction was then quenched for 1 hr by washing in cacodylate buffer containing 0.1 M glycine. A contrast was imparted to the cells by incubation in 1% tannic acid (TA) for 1 hr at 4°C, followed by washing in H_2_O for the second staining of 1% uranyl acetate (aq) for 1 hr at 4°C. *E. coli* cells were dehydrated in an ethanol series (70–100%) for a period of 2 hr for subsequent resin embedding (London Resin Company Ltd., Berkshire, UK).

### 2.5 Ultrathin sectioning of resin blocks for imaging by transmission electron microscopy

Ultrathin (50 nm) sections were cut (using an ultramicrotome [Ultracut E, Reichert‐Jung; Vienna, Austria]) and transferred to 200‐mesh carbon‐coated copper grids (Agar Scientific, Stansted Essex) for immunolabelling of hGH (Section 2.5) and subsequent transmission electron microscopy (TEM) imaging.

### 2.6 Immunolabelling of *E. coli* cells to detect hGH in the inner membrane

Using the touching‐drop method (Rubinstein, 2007), ultrathin sections were blocked via incubation in TBS/Tween buffer (0.24% Tris (w/v), 2.5% NaCl (w/v), and 2% Tween‐20 (v/v)) pH 8.4 containing 1% BSA (w/v) and 4% normal goat sera (v/v) (Abcam Plc, Cambridge, UK) for 30 min at RT. hGH protein was labeled by incubation with primary anti‐hGH antibody (rabbit polyclonal) at 1:20,000 dilution (TBS/Tween + 1% BSA buffer) for 2 hr at RT. Sections were then washed with TBS/Tween + 1% BSA at RT and then incubated with 10 nm gold‐conjugated secondary antibody (Goat antirabbit IgG pre‐adsorbed, Abcam Plc) for 1 hr at RT. Finally, sections were washed in TBS/Tween ( + 1% BSA) and dH_2_O and allowed to air dry before insertion into the electron microscope (EM).

### 2.7 Transmission electron microscopy of ultrathin *E. coli* sections

Ultrathin sections of *E. coli* cells were imaged under a 200 kV JEOL 2010F transmission electron microscope with a field emission gun electron source, operating a Gatan Ultrascan™ 4000 CCD camera with a pixel size of 14 μm.

### 2.8 Quantification of immunogold labeling

A random sampling approach was used to analyze the distribution of gold within the immunolabelled *E. coli* sections, as in [Smith et al., 2017]. An identical Chi‐squared analysis procedure (as in [Smith et al., 2017]) was performed on samples of *E. coli* cells overexpressing either WT or mutant precursor hGH, with the null hypothesis (of no difference in immunolabelling between hGH‐overexpressing cells and WT *E. coli* cells not overexpressing any recombinant protein) being rejected (*p* < 0.005).

### 2.9 Time profile of cell growth of *E. coli* cells overexpressing TorA‐hGH and TorA‐A39L‐hGH

Growth curves of TorA‐hGH and TorA‐A39L‐hGH were generated by growing *E. coli* cells (of WT tat or Δtat background) in LB media. Protein expression was induced at *T* = 0 hr with 1 mM IPTG. OD_600_ values were recorded at 15‐min intervals for the first 3 hr, and then at 1‐hr intervals following this.

### 2.10 TMAO reductase activity assay (TorA assay)

Cells were grown in LB media supplemented with glycerol (0.5% v/v), trimethylamine N‐oxide (TMAO; 0.4% w/v), and sodium molybdate (1 µM; LB‐GT) and fractionated as described in Section 2.2. Serial dilutions of the cytoplasmic and periplasmic fractions (1, 1/10, 1/100, and 1/100) were run on native 12% polyacrylamide gels and the presence of TMAO reductase was detected using a protocol adapted from Silvestro, Pommier, and Giordano (1988). Immediately following native PAGE, the gel was placed in a nitrogen‐saturated container and covered with 100 mM potassium phosphate and 0.25% w/v methyl viologen (buffer saturated with nitrogen gas). The gel was stained blue by adding freshly prepared sodium dithionate (0.1% w/v in 10 mM NaOH) and incubating for 30–60 min until the gel was saturated blue. Gels were then transferred to another container (saturated with nitrogen gas), and covered with 100 mM potassium phosphate and 40 mM TMAO (buffer saturated with nitrogen gas) until white bands could be visualized. Photos were taken on a handheld camera as bands appeared.

### 2.11 Light microscopy of *E. coli* cells

To check for a chain‐forming phenotype of *E. coli* cells overexpressing either TorA‐hGH or TorA‐A39L‐hGH, cells were visualized under a Zeiss Primostar light microscope at X400 magnification.

## 3 RESULTS

### 3.1 TorA‐hGH is exported efficiently by the TAT system

**Figure 1:**
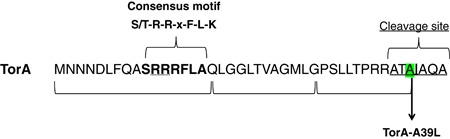
Structure of the TorA signal peptide and the TorA‐A39L variant. Primary structure of the 42‐residue signal peptide of *E. coli* TorA. The consensus motif is highlighted in **bold** (twin‐arginine motif, dotted underline), and the peptidase cleavage site is underlined. The terminal, alanine residue at position 39 (highlighted), was substituted by leucine to generate the TorA‐A39L variant

hGH was expressed with a N‐terminal TorA signal peptide that contains several features typical of a Tat signal peptide (hereafter referred to as TorA‐hGH). These include an N‐terminal domain, a hydrophobic core region and a polar C‐terminal domain ending with an Ala–Xaa–Ala consensus motif (Figure 1). It is this signal peptide that targets hGH, and other proteins, to the inner membrane of *E. coli* for efficient export into the periplasm by the Tat machinery (Ren et al., 2013).

(Alanen et al., 2015) showed that hGH can be exported by Tat and subsequently acquire its disulfide bonds in the periplasm. The aim of this study was to gain a more detailed insight into the export of this biopharmaceutical in *E. coli*, and in particular to directly visualize the location of the exported protein. We initially used standard export assays to examine the export of TorA‐hGH in WT MC4100 *E. coli* cells expressing native levels of Tat machinery. At certain time points, up until 2 hr after the induction of the plasmid, cells were fractionated to generate cytoplasm (C), periplasm (P), and membrane and insoluble (MI) samples which were analyzed by SDS‐PAGE and immunoblotting.

**Figure 2:**
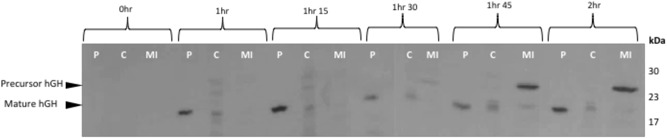
Export assay of Tat substrate (TorA‐hGH) into the periplasm of *E. coli*. Western blot to detect the presence of hGH in *E. coli*. Postinduction with 1 mM IPTG *E. coli* cells overexpressing hGH fused to a TorA signal peptide (TorA‐hGH) were grown at 37°C for 2 hr. During this time, cells were periodically harvested, normalized for OD_600_ = 10 and subsequently fractionated to periplasm (P), cytoplasm (C), and membrane/insoluble fractions (MI) fractions. Each fraction was examined for the presence of hGH by immunoblotting with anti‐hGH antibody. Results show there is a good level of export from as early as 1 hr after induction, shown by the presence of mature hGH (22 kDa) in the periplasm. At each time point there is a lack of precursor (27 kDa) TorA‐hGH (i.e., Tat substrate) in the cytoplasmic fractions, indicating the substrate is either degraded or forming inclusion bodies in the cell. From 1 hr 45 min onwards, there is a consistent level of precursor TorA‐hGH in the membrane fraction of the *E. coli* cells suggesting to the formation of inclusion bodies. hGH, human growth hormone; IPTG, isopropyl β‐D‐1‐thiogalactopyranoside

Immunoblotting against hGH protein showed two forms of hGH are produced in whole cell fractions: a 27 kDa precursor protein and 22 kDa mature protein (Figures 2, 4). Some nonspecific bands are also observed.

**Figure 3:**
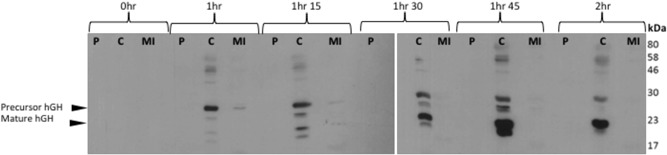
Export assay of TorA‐hGH into the periplasm of *E. coli* cells lacking expression of Tat machinery western blot to detect the presence of hGH in *E. coli*. Postinduction with 1 mM IPTG *E. coli* cells overexpressing hGH fused to a TorA signal peptide (TorA‐hGH) and lacking expression of Tat machinery, that is, ∆*tat* were grown at 37°C for 2 hr. During this time, cells were periodically harvested, normalized for OD_600_ = 10 and subsequently fractionated to periplasm (P), cytoplasm (C), and membrane/insoluble fractions (MI) fractions. Each fraction was examined for the presence of hGH by immunoblotting with anti‐hGH antibody. Results show that mature hGH (22 kDa) is present in the cytoplasm from very early on (1 hr). Precursor hGH (27 kDa) expression increases at 1 hr 30, and there is no hGH present in the MI fraction at all time points tested. hGH, human growth hormone; IPTG, isopropyl β‐D‐1‐thiogalactopyranoside

**Figure 4:**
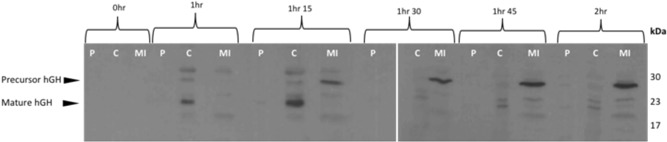
Export assay of a noncleavable Tat substrate (TorA‐A39L‐hGH) into the periplasm of *E. coli*. Western blot to detect the presence of noncleavable hGH in *E. coli*. Postinduction with 1 mM IPTG *E. coli* cells overexpressing hGH fused to a noncleavable TorA signal peptide (TorA‐A39L‐hGH) were grown at 37°C for 2 hr. During this time, cells were periodically harvested, normalized for OD_600_ = 10 and subsequently fractionated to periplasm (P), cytoplasm (C), and membrane/insoluble (MI) fractions. Each fraction was examined for the presence of hGH by immunoblotting with anti‐hGH antibody. A lack of hGH protein in the periplasm of any time point confirms that the Tat substrate is noncleavable and thus remains in the inner membrane fraction. hGH is degraded in the cytoplasm from as early as 1 hr indicated by the mature sized hGH (22 kDa). This is degraded completely by 1 hr 30 min. From 1 hr 15 min onwards there is a consistent level of precursor TorA‐hGH (27 kDa) in the membrane fraction of the *E. coli*. hGH, human growth hormone; IPTG, isopropyl β‐D‐1‐thiogalactopyranoside

Export assays demonstrated that Tat‐mediated transport of TorA‐hGH occurs soon after the induction since, after 1 hr, mature hGH is present in the periplasm. There is a minimal amount of precursor protein present in the cytoplasmic fraction, which has also been observed in previous analyses of Tat‐dependent export of TorA‐hGH (Ren et al., 2013). Therefore, the lack of cytoplasmic precursor protein in this study could be due to the quick turnover of the substrate from the cytoplasm to the periplasm, or due to proteolytic cleavage of the precursor protein (Figure 2). Quick turnover of the precursor hGH would also explain the lack and/or minimal amount of precursor hGH present at the membrane up until 1 hr 30. Only at later time points (1 hr 45 and 2 hr) do we see a substantial increase in precursor at the membrane, which is consistent with increased production of hGH precursor/Tat substrate.

To confirm that the export of TorA‐hGH is via Tat, an identical export assay was performed in *E. coli* cells lacking Tat machinery (Δ*tatABCDE*; hereafter denoted Δ*tat*). The lack of mature hGH in the periplasm of these control cells, at any time point, confirms Tat‐dependent export of hGH into the periplasm in WT *E. coli* (Figure 3).

### 3.2 Substitution of the −1 position of the signal peptide blocks maturation of TorA‐hGH

It has previously been shown that the nature of the amino acid side chain at the −1 position of the Tat signal peptide (i.e., the last residue of the signal peptide) is essential for efficient maturation of a Tat precursor protein (Ren et al., 2013). The maturation enzyme, leader peptidase, will only tolerate a short chain residue such as alanine at this position. Substitution of the alanine residue at the −1 position of the signal peptide by leucine completely blocked maturation of a native Tat substrate, YedY, with the protein remaining membrane‐bound and its mature domain exposed to the periplasm (Ren et al., 2013).

To test whether the same mutation blocks maturation of hGH—a biotechnologically relevant, heterologous Tat substrate—export assays were conducted as in Section 3.1, with the −1 alanine residue in the TorA signal peptide of TorA‐hGH substituted with leucine (generating TorA‐A39L‐hGH).

Consistent with the findings of (Alanen et al., 2015; Ren et al., 2013), the precursor protein was not processed to any significant degree and is found almost exclusively in the membrane fraction as a precursor protein (Figure 4; 1 hr 15 time point onwards). As seen with the WT precursor, mature hGH was detected in the cytoplasm from an early time point (1 hr), which most likely represents proteolytic cleavage of the signal peptide, as observed previously (Alanen et al., 2015).

To confirm that the results of our export assays are not affected by inaccurate fractionation of *E. coli*, we immunoblotted for the presence of maltose binding protein and LacI in the cytoplasm, membrane and insoluble, and periplasm fractions (Supporting Information Figure S1). LacI (which can be sequestered to the inner membrane as a means of gene regulation in bacteria [Görke, Reinhardt, & Rak, 2005]) was not detected in the periplasm (Supporting Information Figure S1, top panel), confirming the successful isolation of the periplasmic region from the cytoplasmic and membrane/insoluble fractions with no/negligible contamination. Detection of maltose binding protein in all three fractions of *E. coli* (particularly with the least amount in the membrane and insoluble fraction; Supporting Information Figure S1, bottom panel) is consistent with the transient export of this protein into the periplasm via the Sec translocase machinery. Together, these data confirm the accurate and reproducible fractionation of *E. coli* cells used in this study.

#### 3.2.1 Expression of TorA‐A39L‐hGH does not affect *E. coli* viability

To confirm that the mutated TorA signal peptide (A39L) does not cause permanent blockage of the Tat machinery, which would otherwise cause a problem with cell viability, a growth curve of *E. coli* cells (overexpressing either TorA‐hGH or TorA‐A39L‐hGH) was conducted. Results show that the growth profile of *E. coli* is near‐identical when overexpressing either TorA‐hGH or TorA‐A39L‐hGH (Supporting Information Figure S2).

#### 3.2.2 Expression of TorA‐A39L‐hGH does not block translocation of TMAO reductase

It is important to understand if normal functioning of the twin‐arginine translocase occurs in cells expressing a substrate that is blocked in the membrane. To this end, we used an assay for a native Tat substrate TorA (TMAO reductase). TorA is a large (90 kDa), cofactor binding enzyme of *E. coli* that is transported to the periplasm via the Tat system after binding two molybdenum guanine dinucleotide (MGD) molecules in the cytoplasm. It is responsible for the reduction of the terminal electron acceptor TMAO to trimethylamine (Barrett & Kwan, 1985). TorA has enzymatic activity in both the cytoplasm and the periplasm (Sargent et al., 1998) and is, therefore, an ideal candidate to show whether translocation is blocked.

**Figure 5:**
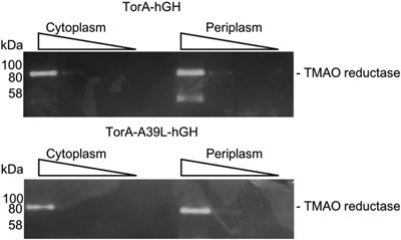
The endogenous Tat substrate TMAO reductase is correctly localized in cells expressing a noncleavable Tat substrate (TorA‐A39‐hGH). *E. coli* MC4100 were grown for 3 hr in LB‐GT media expressing either TorA‐hGH (top panel) or TorA‐A39L‐hGH (bottom panel) and then fractionated in cytoplasm and periplasm. These fractions were serially diluted (decreasing concentration indicated by slope) and run on native PAGE. Gels were then stained for TMAO reductase activity. For cells expressing TorA‐hGH (top panel), TMAO reductase activity can be seen in both the cytoplasm and periplasm. hGH, human growth hormone; PAGE, polyacrylamide gel electrophoresis; TMAO, trimethylamine N‐oxide

To conduct the assay cells are grown in LB‐GT media and either TorA‐hGH or TorA‐A39L‐hGH were expressed for 3 hr. The cells were then fractionated into cytoplasm, membrane, and periplasm, and serial dilutions of cytoplasm and periplasm were separated on native polyacrylamide gels. The gels were stained with reduced methyl viologen before being incubated with TMAO in an anaerobic environment. The appearance of white bands on the dark background indicates the activity, and thus localization of TMAO reductase through the coupled reduction of TMAO and oxidation of methyl viologen. The results of the TMAO reductase assay are shown in Figure 5. In cells expressing TorA‐hGH TMAO reductase activity can be seen in both the cytoplasm and periplasm indicating normal functioning of the Tat system. The lower panel shows TMAO reductase is also active in the periplasm of cells expressing TorA‐A39L‐hGH confirming the mutation that blocks this substrate in the membrane does not affect the ability of the Tat system to translocate other Tat‐dependent proteins.

#### 3.2.3 Expression of TorA‐A39L‐hGH does not cause a chain‐forming phenotype

**Figure 6:**
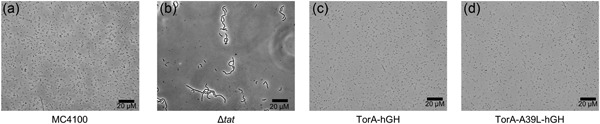
Expression of a noncleavable Tat substrate (TorA‐A39L‐hGH) does not block translocation AmiA and AmiC. Left to right: Microscope images of empty MC4100, empty MC4100 *Δtat*, MC4100 TorA‐hGH, and MC4100 TorA‐A39L‐hGH. The *Δtat* image clearly shows the chain‐forming phenotype. Images of cells expressing TorA‐hGH and TorA‐A39L‐hGH resemble that of the empty wild‐type. Scale is bar 20 μM. X400 magnification. hGH, human growth hormone

Previous studies have shown that inactivation of the *E. coli* Tat pathway causes defects in cell division. This manifests as the formation of cell‐chains, visible under a light microscope (Stanley, Findlay, Berks, & Palmer, 2001). These chains, up to 10‐cells long, are likely caused by the inability to correctly localize the native Tat substrates AmiA and AmiC. AmiA and AmiC are amidases involved in murein cleavage during cell division and naturally reside in the periplasm. Empty MC4100 cells expressing either TorA‐hGH or TorA‐A39L‐hGH, and MC4100 Δ*tat* were grown aerobically for 3 hr. Images representative of these cultures is shown in Figure 6. Empty MC4100 cells are clearly shown as individual cells (Figure 6a). For contrast, panel B of Figure 6 shows “cell chains” that are formed when the Tat machinery is inhibited (or in this case removed). Panels C and D show cultures expressing TorA‐hGH and TorA‐A39L‐hGH, respectively, and these display a phenotype like that of the empty MC4100 (Figure 6a). These images help to confirm the Tat substrate blocked in the membrane does not hinder the export of other Tat‐targeted substrates.

### 3.3 Direct visualization of a biotherapeutic, hGH, in *E. coli*

#### 3.3.1 Visualization of TorA‐hGH

Harnessing of industrial microorganisms, such as *E. coli*, for the production of desired products is reliant on the efficient performance of these “cell factories.” As such, numerous factors serve as important indicators of platform fitness including: protein localization and distribution, and cellular morphology. Previous studies that have analyzed the export of biotechnologically relevant proteins by the Tat machinery have primarily used biochemical approaches (Karlsson et al., 2012; Ren et al., 2013), yet direct visualization of the microorganism, in this case *E. coli*, could provide novel insight into the export of such substrates. In this study, we analyzed the location of TorA‐hGH to test whether the exported protein is indeed randomly distributed in the periplasm, and focused particular attention on the noncleavable A39L‐hGH to determine whether the protein is uniformly distributed in the inner membrane.

To do this, *E. coli* cells overexpressing TorA‐hGH or TorA‐A39L‐hGH were fixed and stained. 2D sections of individual *E. coli* cells were then cut, immunolabelled and finally examined for the presence of gold particles. Use of antibodies specific to hGH avoids the use of (potentially large) affinity tags, and thus permits direct protein detection. Gold particles were classified according to their location, and for the present study, a gold particle found within 25 nm of the inner membrane was defined as being located at the inner membrane. Our aim was to achieve unambiguous identification of the hGH protein in the inner membrane through immunogold labeling of *E. coli* cells overexpressing TorA‐hGH or TorA‐A39L‐hGH. To do this, the level of nonspecific antibody labeling was assessed through comparison of hGH‐overexpressing cells with MC4100 *E. coli* cells that did not overexpress any recombinant protein.

**Figure 7:**
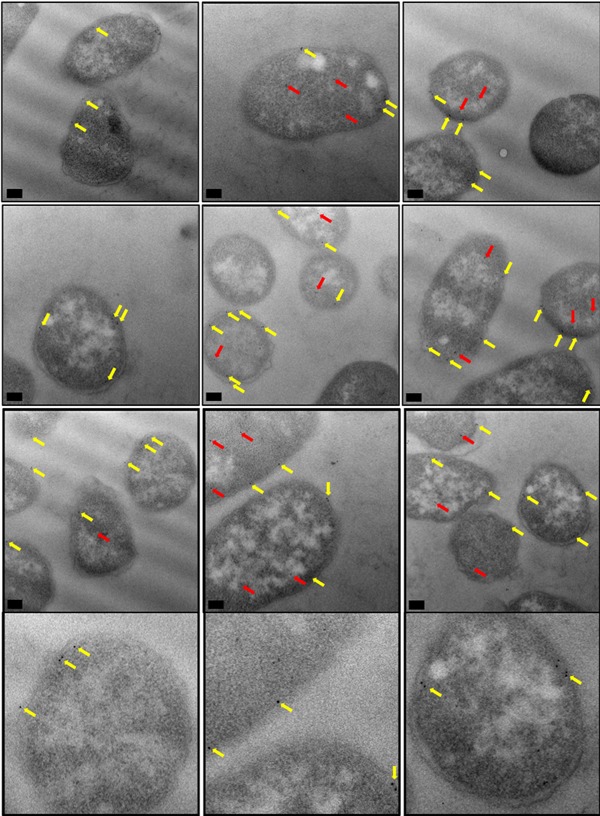
Electron micrographs of *E. coli* cells, overexpressing TorA‐hGH (WT precursor), immunogold labeled following primary antibody detection against hGH protein. Ultrathin sections of *E. coli* cells overexpressing TorA‐hGH (WT precursor) were immunolabelled using a polyclonal antibody raised against hGH (shown in rows 1–3, with row 4 showing closeups of individual gold particles from row 3). At 1 hr 45 min after induction, hGH was found to exhibit a random distribution in the inner membrane (yellow arrows) and was also present in the cytoplasm (red arrows). Images were taken on a JEOL 2010F at X15,000 magnification. Scale bar = 200 nm. hGH, human growth hormone; WT, wild type

Figure 7 shows representative images of TorA‐hGH‐overexpressing cells that were immunogold labeled with anti‐hGH. These cells exhibit a pattern of gold labeling that is predominantly around the periphery of the cell. This was confirmed at increased magnification, as in Figures 6 (bottom row), where the gold particles could be seen to reside within 25 nm of the inner membrane of the cell wall. Given that the bulk of the protein has been shown to be present in periplasmic fractions using export assays (Figure 2), we conclude that these particles are indeed located in the periplasm of the cells. A smaller number of particles were present in the cytoplasm. Magnified images of these cells, with closeups of individual gold particles, can also be seen in Supporting Information Figure S3.

Analysis of *E. coli* cells that did not express TorA‐hGH (representative images shown in Supporting Information Figure S4), showed the presence of only a few gold particles in the cytoplasmic and membranous regions. These data indicate that the antibody is able to detect hGH with a high degree of specificity. As further controls, TorA‐hGH‐expressing cells were immunolabelled with the primary antibody omitted (Supporting Information Figure S5, a and b), and the TEM analysis showed that these cells lacked any gold binding confirming that nonspecific binding seen in *E. coli* cells not overexpressing any recombinant protein was attributable to the primary antibody. Finally, the same cell type was immunolabelled with a gold‐conjugated secondary antibody directed towards a different animal species (Supporting Information Figure S5, c and d). The TEM analysis showed only unlabeled *E. coli* cells, confirming that the cells do not have a nonspecific attraction for gold particles.

**Figure 8:**
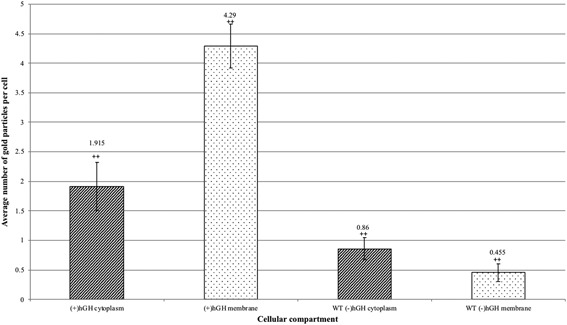
Quantitative analysis of raw gold counts of *E. coli* cells immunogold labeled with primary antibody detection against TorA‐hGH protein. *E. coli* cells overexpressing TorA‐hGH were immunolabelled using a primary antibody against the hGH protein. Controls were *E. coli* cells that lacked expression of hGH protein (labeled (‐) hGH). Raw gold counts were taken from 200 randomly imaged *E. coli* from two separate resin blocks. Gold was assigned to either “membrane + periplasm” (labeled membrane) or cytoplasm compartment. The approximate numbers of gold particles at the cytoplasm and membrane fraction of each cell were calculated for each cell type. There was 2.2x more gold in the cytoplasm (line‐patterned) of hGH expressing cells versus those lacking hGH expression. There was 9.43x more gold in the membrane (dotted pattern) of hGH expressing cells versus those lacking hGH expression. Error bars: CI of 2x SE. The labeling of hGH between the two cell types is statistically significant (indicated by ++). hGH, human growth hormone

To quantitate the degree of specific labeling, raw gold counts were collected from 200 randomly sampled, immunolabelled *E. coli* with or without the overexpression of TorA‐hGH, and the gold particles were assigned to either the cytoplasmic or inner membrane/periplasm compartments. Quantification of the gold particles (Figure 8) shows that the average number of gold particles per compartment in TorA‐hGH‐overexpressing cells was 1.92 per cell ( ± 0.41 gold) in the cytoplasm and 4.29 per cell ( ± 0.37 gold) in the inner membrane, whereas *E. coli* cells lacking overexpression of TorA‐hGH bound 0.86 per cell in the cytoplasm ( ± 0.19 gold) and 0.46 ( ± 0.15 gold) in the membrane, respectively. The labeling of membrane‐bound TorA‐hGH is thus highly specific, with the inner membrane/periplasm of TorA‐hGH‐overexpressing cells containing 9.42‐fold more gold particles than the same membrane in *E. coli* cells that lack overexpression of TorA‐hGH. To confirm a statistical independence in immunogold labeling between these cells at the cytoplasm and inner membrane, χ^2^ analysis of the raw gold count data was conducted. For a total χ^2^ value of 111.2 and 1 degree of freedom, *p* was < 0.005. This confirms that the gold labeling distributions between the two cell types are significantly different.

Analysis of the distribution of gold particles in the cytoplasm shows the presence of 1.92 and 0.86 particles in the TorA‐hGH‐overexpressing and non‐hGH expressing *E. coli* cells, respectively. Since the number of gold particles in *E. coli* cells that lack overexpression of hGH represents nonspecific binding, and assuming that the level of nonspecific binding in the two cell types is the same, this indicates that TorA‐hGH‐overexpressing cells contain an average of one gold particle per cell in the cytoplasm, which is considerably lower than the 3.8 gold particles per cell in the inner membrane/periplasm region. This difference is consistent with the amount of hGH in the cytoplasmic fraction compared with the membranous compartment of *E. coli* determined by export assays, which revealed that at 1 hr 45 min after induction, there is considerably more hGH present in the membrane fraction compared with the cytoplasm (Figure 2). Our immunolabelling approach thus provides highly specific detection of hGH in both the cytoplasmic and membrane fractions of *E. coli*.

Immunogold labeling of TorA‐hGH showed this protein is randomly distributed within the *E. coli* periplasm and cytoplasm, with no evidence for a preferential localization at the poles or elsewhere (Figure 7, yellow and red arrows, respectively). This is the first time that a heterologous Tat substrate has been directly visualized after export to the periplasm, and these observations are consistent with the efficient production of hGH in our *E. coli* cells.

#### 3.3.2 Visualization of noncleavable TorA‐hGH

**Figure 9:**
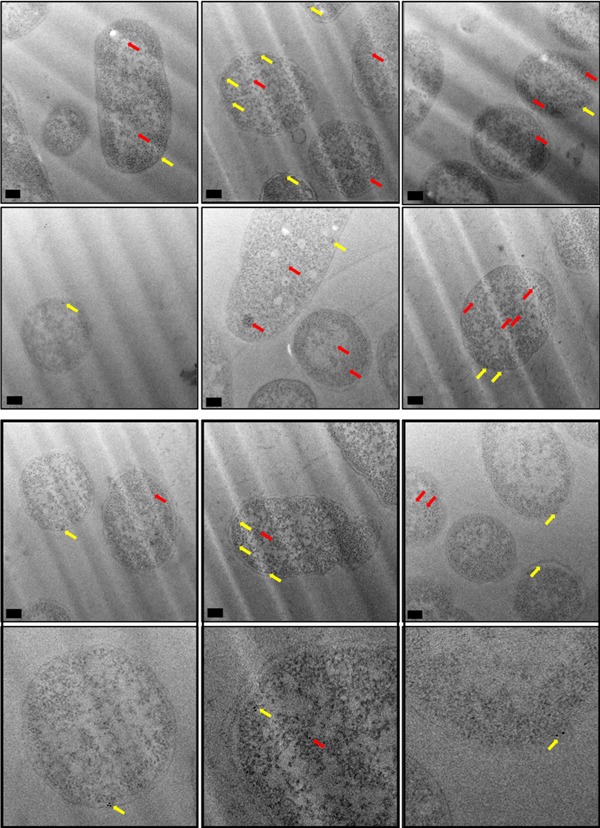
Electron micrographs of *E. coli* cells, overexpressing TorA‐hGH (mutant precursor), immunogold labeled following primary antibody detection against hGH protein. Ultrathin sections of *E. coli* cells overexpressing TorA‐hGH (mutant precursor) were immunolabelled using a polyclonal antibody raised against hGH (shown in rows 1–3, with row 4 showing closeups of individual gold particles from row 3). At 1 hr 45 min after induction, hGH was found to exhibit a random distribution in the inner membrane (yellow arrows) and was also present in the cytoplasm (red arrows). Images were taken on a JEOL 2010F at X15,000 magnification. Scale bar = 200 nm. hGH, human growth hormone

To investigate whether a similar distribution and/or localization occurs for the mutated TorA‐hGH construct, (TorA‐A39L‐hGH), we immunogold labeled *E. coli* cells overexpressing TorA‐A39L‐hGH to detect the presence of hGH. Analysis of the localization and distribution of gold particles showed that similar to the WT precursor (TorA‐hGH, Section 3.3.1), TorA‐A39L‐hGH exhibits a random distribution in the cytoplasm (red arrows) and a uniform distribution in the inner membrane (yellow arrows) of the *E. coli* cell (Figure 9). This was confirmed at higher magnifications, as in Figures 8 (bottom row) and Supporting Information Figure S6. Therefore, in addition to biochemical export assays (Figure 4) which showed a lack of TorA‐A39L‐hGH in the periplasm, these EM data provide direct evidence that an unprocessed Tat precursor remains membrane‐associated despite being unable to undergo cleavage by signal peptidases in the periplasm.

## 4 DISCUSSION

The twin‐arginine translocase offers the potential for the biotechnology industry owing to its ability to transport fully‐folded proteins into the periplasm of *E. coli*. Initial studies confirmed the capability of the Tat system to transport a model heterologous protein to a high yield (Matos et al., 2012), and more recent data has shown that substrates are not limited to GFP with Tat being able to transport hGH, scFv, and interferon α2b (Alanen et al., 2015; Browning et al., 2017). Furthermore, it has also been shown that it is possible to anchor a Tat substrate in the inner membrane of *E. coli* with the mature domain of the protein facing the periplasmic side of the membrane (Karlsson et al., 2012; Ren et al., 2013). Combined, these data reveal a novel potential for the Tat system in producing single‐span membrane proteins in *E. coli*.

Anchoring of heterologous Tat substrates in the inner membrane has previously occurred via the addition of extra amino acid residues to the protein sequence (Karlsson et al., 2012). However, this study has shown that only a single substitution mutation in the signal peptidase cleavage site of a TorA signal peptide, fused to hGH, resulted in the precursor protein being “stalled” at the inner membrane, with no alterations to the mature protein. The previous studies by (Karlsson et al., 2012) and (Ren et al., 2013) showed that cleavage by leader peptidase occurs only after transfer of the mature protein to the periplasm, and given that hGH is exported by Tat in an extremely efficient manner, it is to be expected that the mature hGH protein will be similarly positioned on the periplasmic face of the inner membrane. This could be confirmed using a structural biology approach whereby *E. coli* spheroblasts (overexpressing TorA‐hGH or TorA‐A39L‐hGH) are immunogold labeled specifically for hGH and subsequently visualized by SEM. This would allow visualization of the periplasmic side of the inner membrane with the hGH‐bound immunogold, and confirm the topology of the mature hGH as periplasmically exposed.

In summary, a combination of biochemical export assays and imaging approaches were used in this study to investigate the temporal expression of TorA‐hGH and TorA‐A39L‐hGH in *E. coli* and to determine whether they were transported via Tat machinery. Our data serves as a proof‐of‐concept study where we have demonstrated the Tat‐dependent transport of TorA‐hGH into the periplasm and most importantly, the stable expression of TorA‐A39L‐hGH at the inner membrane. We propose that this novel methodology could be developed for the targeting, transport, and display of other industrially important single‐span membrane proteins at the inner membrane of *E. coli* via the Tat machinery. Having shown the potential of this system for the display of one industrially relevant target protein, hGH, we acknowledge that further studies with a more tractable substrate protein would be of interest to enable activity on the inner membrane to be determined. This further work would develop our novel methodology and fully explore its application for other targets.

## CONFLICTS OF INTEREST

The authors declare that there are no conflicts of interest associated with the manuscript.

## Supporting information

Supporting information

Supporting information

Supporting information

Supporting information

Supporting information

Supporting information

## References

[bit26895-bib-0001] Alanen, H. I. , Walker, K. L. , Lourdes Velez Suberbie, M. , Matos, C. F. R. O. , Bönisch, S. , Freedman, R. B. , … Robinson, C. (2015). Efficient export of human growth hormone, interferon α2b and antibody fragments to the periplasm by the *Escherichia coli* Tat pathway in the absence of prior disulfide bond formation. Biochimica et Biophysica Acta (BBA) ‐ Molecular Cell Research, 1853(3), 756–763. 25554517 10.1016/j.bbamcr.2014.12.027

[bit26895-bib-0002] Barrett, E. L. , & Kwan, H. S. (1985). Bacterial reduction of trimethylamine oxide. Annual Review of Microbiology, 39(1), 131–149. 10.1146/annurev.mi.39.100185.001023 3904597

[bit26895-bib-0003] Browning, D. F. , Richards, K. L. , Peswani, A. R. , Roobol, J. , Busby, S. J. W. , & Robinson, C. (2017). *Escherichia coli* “TatExpress” strains super‐secrete human growth hormone into the bacterial periplasm by the Tat pathway. Biotechnology and Bioengineering, 114(12), 2828–2836. 28842980 10.1002/bit.26434 PMC5698719

[bit26895-bib-0004] Chen, W. , & Georgiou, G. (2002). Cell‐surface display of heterologous proteins: From high‐throughput screening to environmental applications. Biotechnology and Bioengineering, 79(5), 496–503. 12209821 10.1002/bit.10407

[bit26895-bib-0005] DeLisa, M. P. , Tullman, D. , & Georgiou, G. (2003). Folding quality control in the export of proteins by the bacterial twin‐arginine translocation pathway. Proceedings of the National Academy of Sciences of the United States of America, 100(10), 6115–6120. 12721369 10.1073/pnas.0937838100 PMC156335

[bit26895-bib-0006] Driessen, A. J. M. , & Nouwen, N. (2008). Protein translocation across the bacterial cytoplasmic membrane. Annual Review of Biochemistry, 77(1), 643–667. 10.1146/annurev.biochem.77.061606.160747 18078384

[bit26895-bib-0007] Fisher, A. C. , Kim, W. , & DeLisa, M. P. (2006). Genetic selection for protein solubility enabled by the folding quality control feature of the twin‐arginine translocation pathway. Protein Science: A Publication of the Protein Society, 15(3), 449–458. 16452624 10.1110/ps.051902606 PMC2249766

[bit26895-bib-0008] Görke, B. , Reinhardt, J. , & Rak, B. (2005). Activity of Lac repressor anchored to the *Escherichia coli* inner membrane. Nucleic Acids Research, 33(8), 2504–2511. 15867195 10.1093/nar/gki549 PMC1088070

[bit26895-bib-0009] Karlsson, A. J. , Lim, H. ‐K. , Xu, H. , Rocco, M. A. , Bratkowski, M. A. , Ke, A. , & DeLisa, M. P. (2012). Engineering antibody fitness and function using membrane‐anchored display of correctly folded proteins. Journal of Molecular Biology, 416(1), 94–107. 22197376 10.1016/j.jmb.2011.12.021 PMC3268853

[bit26895-bib-0010] Kassem, M. , Blum, W. , Ristelli, J. , Mosekilde, L. , & Eriksen, E. F. (1993). Growth hormone stimulates proliferation and differentiation of normal human osteoblast‐like cells in vitro. Calcified Tissue International, 52(3), 222–226. 7683248 10.1007/BF00298723

[bit26895-bib-0011] Matos, C. F. , Branston, S. D. , Albiniak, A. , Dhanoya, A. , Freedman, R. B. , Keshavarz‐Moore, E. , & Robinson, C. (2012). High‐yield export of a native heterologous protein to the periplasm by the tat translocation pathway in *Escherichia coli* . Biotechnology & Bioengineering, 109(10), 2533–2542. 22539025 10.1002/bit.24535

[bit26895-bib-0012] Matos, C. F. R. O. , Robinson, C. , Alanen, H. I. , Prus, P. , Uchida, Y. , Ruddock, L. W. , … Keshavarz‐Moore, E. (2013). Efficient export of prefolded, disulfide‐bonded recombinant proteins to the periplasm by the Tat pathway in *Escherichia coli* CyDisCo strains. Biotechnology Progress, 30(2), 281–290. 10.1002/btpr.1858 24376243

[bit26895-bib-0013] Mazor, Y. , Van Blarcom, T. , Iverson, B. L. , & Georgiou, G. (2008). E‐clonal antibodies: Selection of full‐length IgG antibodies using bacterial periplasmic display. Nature Protocols, 3, 1766–1777. 18948976 10.1038/nprot.2008.176

[bit26895-bib-0014] Moghaddam‐Taaheri, P. , Ikonomova, S. P. , Gong, Z. , Wisniewski, J. Q. , & Karlsson, A. J. (2016). Bacterial inner‐membrane display for screening a library of antibody fragments. Journal of Visualized Experiments: JoVE, 116, 54583. 10.3791/54583 PMC5092199 27805609

[bit26895-bib-0015] Pooley, H. M. , Merchante, R. , & Karamata, D. (1996). Overall protein content and induced enzyme components of the periplasm of *Bacillus subtilis* . Microbial Drug Resistance, 2(1), 9–15. 9158717 10.1089/mdr.1996.2.9

[bit26895-bib-0016] Randall, L. L. , & Hardy, S. J. S. (1986). Correlation of competence for export with lack of tertiary structure of the mature species: A study in vivo of maltose‐binding protein in *E. coli* . Cell, 46(6), 921–928. 3530497 10.1016/0092-8674(86)90074-7

[bit26895-bib-0017] Ren, C. , Patel, R. , & Robinson, C. (2013). Exclusively membrane‐inserted state of an uncleavable Tat precursor suggests lateral transfer into the bilayer from the translocon. FEBS Journal, 280(14), 3354–3364. 23647663 10.1111/febs.12327

[bit26895-bib-0018] Rubinstein, J. L. (2007). Structural analysis of membrane protein complexes by single particle electron microscopy. Methods, 41(4), 409–416. 17367713 10.1016/j.ymeth.2006.07.019

[bit26895-bib-0019] Sanchez‐Ortiga, R. , Klibanski, A. , & Tritos, N. A. (2012). Effects of recombinant human growth hormone therapy in adults with Prader‐Willi syndrome: A meta‐analysis. Clinical Endocrinology, 77(1), 86–93. 22117629 10.1111/j.1365-2265.2011.04303.x

[bit26895-bib-0020] Sargent, F. , Bogsch, E. G. , Stanley, N. R. , Wexler, M. , Robinson, C. , Berks, B. C. , & Palmer, T. (1998). Overlapping functions of components of a bacterial Sec‐independent protein export pathway. The EMBO Journal, 17(13), 3640–3650. 9649434 10.1093/emboj/17.13.3640 PMC1170700

[bit26895-bib-0021] Silvestro, A. , Pommier, J. , & Giordano, G. (1988). The inducible trimethylamine‐N‐oxide reductase of *Escherichia coli* K12: Biochemical and immunological studies. Biochimica et Biophysica Acta (BBA) ‐ Protein Structure and Molecular Enzymology, 954, 1–13. 3282544 10.1016/0167-4838(88)90049-0

[bit26895-bib-0022] Smith, S. M. , Yarwood, A. , Fleck, R. A. , Robinson, C. , & Smith, C. J. (2017). TatA complexes exhibit a marked change in organisation in response to expression of the TatBC complex. Biochemical Journal, 474(9), 1495–1508. 28280110 10.1042/BCJ20160952 PMC5396077

[bit26895-bib-0023] Spiliotis, B. (2008). Recombinant human growth hormone in the treatment of Turner syndrome. Therapeutics and Clinical Risk Management, 4(6), 1177–1183. 19337425 10.2147/tcrm.s1440 PMC2643099

[bit26895-bib-0024] Stanley, N. R. , Findlay, K. , Berks, B. C. , & Palmer, T. (2001). *Escherichia coli* Strains blocked in Tat‐dependent protein export exhibit pleiotropic defects in the cell envelope. The Journal of Bacteriology, 183(1), 139–144. 11114910 10.1128/JB.183.1.139-144.2001 PMC94859

[bit26895-bib-0025] Takeda, A. , Cooper, K. , Bird, A. , Baxter, L. , Frampton, G. , Gospodarevskaya, E. , … Bryant, J. (2010). Recombinant human growth hormone for the treatment of growth disorders in children: A systematic review and economic evaluation. Health Technology Assessment, 14(42), 1–209. 10.3310/hta14420 20849734

[bit26895-bib-0026] Vogt, K. , & Emerick, J. (2015). Growth hormone therapy in adults with Prader‐Willi syndrome. Diseases, 3, 56–67. 28943608 10.3390/diseases3020056 PMC5548233

[bit26895-bib-0027] Walsh, G. (2010). Post‐translational modifications of protein biopharmaceuticals. Drug Discovery Today, 15(17–18), 773–780. 20599624 10.1016/j.drudis.2010.06.009

[bit26895-bib-0028] Wu, C. H. , Mulchandani, A. , & Chen, W. (2008). Versatile microbial surface‐display for environmental remediation and biofuels production. Trends in Microbiology, 16(4), 181–188. 18321708 10.1016/j.tim.2008.01.003

