# Supplementary material for: Characterization of a novel method for the production of single‐span membrane proteins in Escherichia coli
Source: Biotechnol Bioeng. 2019 Jan 19;116(4):722–33. doi: 10.1002/bit.26895 (PMC6492203; doi:10.1002/bit.26895)
Supplement: Supplementary file 1 — Supporting information [file BIT-116-722-s001.pdf]

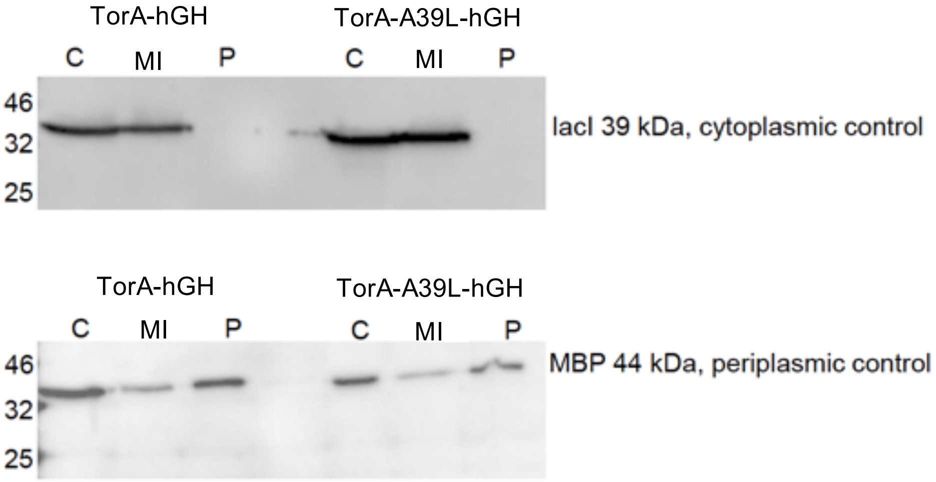

Figure S1. Immunodetection of LacI and Maltose Binding Protein in *E. coli* cells overexpressing TorA-hGH or TorA-A39L-hGH

Western Blot to detect the presence of LacI and maltose binding protein (MBP) in *E. coli*. Post-induction with 1 mM IPTG *E. coli* cells overexpressing hGH fused to a TorA signal peptide (TorA-hGH) or hGH fused to a non-cleavable TorA signal peptide (TorA-A39L-hGH) were grown at 37°C for 2 hours. After this time, cells were harvested, normalised for OD_{600} = 10 and subsequently fractionated to cytoplasm (C), membrane/insoluble fractions (MI) and periplasmic (P) fractions. Each fraction was examined for the presence of LacI and MBP by immunoblotting with anti-LacI and anti-MBP antibody, respectively. Top panel: results show that LacI was not detected in the periplasm of *E. coli* cells overexpressing either TorA-hGH or TorA-A39L-hGH, but present in C and MI fractions. Bottom panel: MBP was detected in all three fractions, notably with the least amount in the MI fraction. The subcellular location of LacI and MBP confirms the reliable fractionation of *E. coli* cells used in this study.
