# Supplementary material for: Characterization of a novel method for the production of single‐span membrane proteins in Escherichia coli
Source: Biotechnol Bioeng. 2019 Jan 19;116(4):722–33. doi: 10.1002/bit.26895 (PMC6492203; doi:10.1002/bit.26895)
Supplement: Supplementary file 2 — Supporting information [file BIT-116-722-s002.pdf]

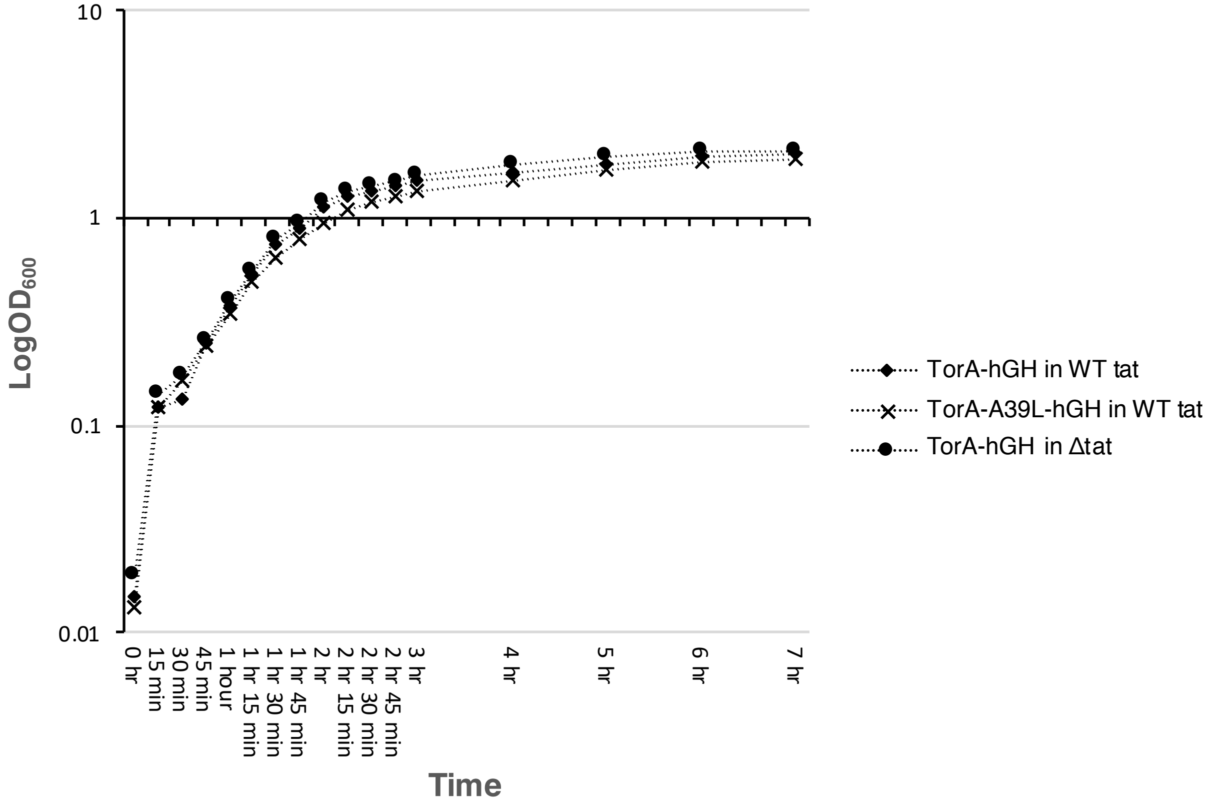

Figure S2. Time profile of cell growth for the hGH constructs used in this study. Growth curves of TorA-hGH and TorA-A39L-hGH were generated by growing *E. coli* cells (of WT tat or Δtat background) in Luria Broth media. Protein expression was induced at T=0 hr with 1 mM IPTG. OD_{600} values were recorded at 15 minute intervals for the first 3 hours, and then at 1 hour intervals following this. The growth profile of *E. coli* is near-identical when overexpressing either TorA-hGH or TorA-A39L-hGH.
