# Supplementary material for: Characterization of a novel method for the production of single‐span membrane proteins in Escherichia coli
Source: Biotechnol Bioeng. 2019 Jan 19;116(4):722–33. doi: 10.1002/bit.26895 (PMC6492203; doi:10.1002/bit.26895)
Supplement: Supplementary file 3 — Supporting information [file BIT-116-722-s003.pdf]

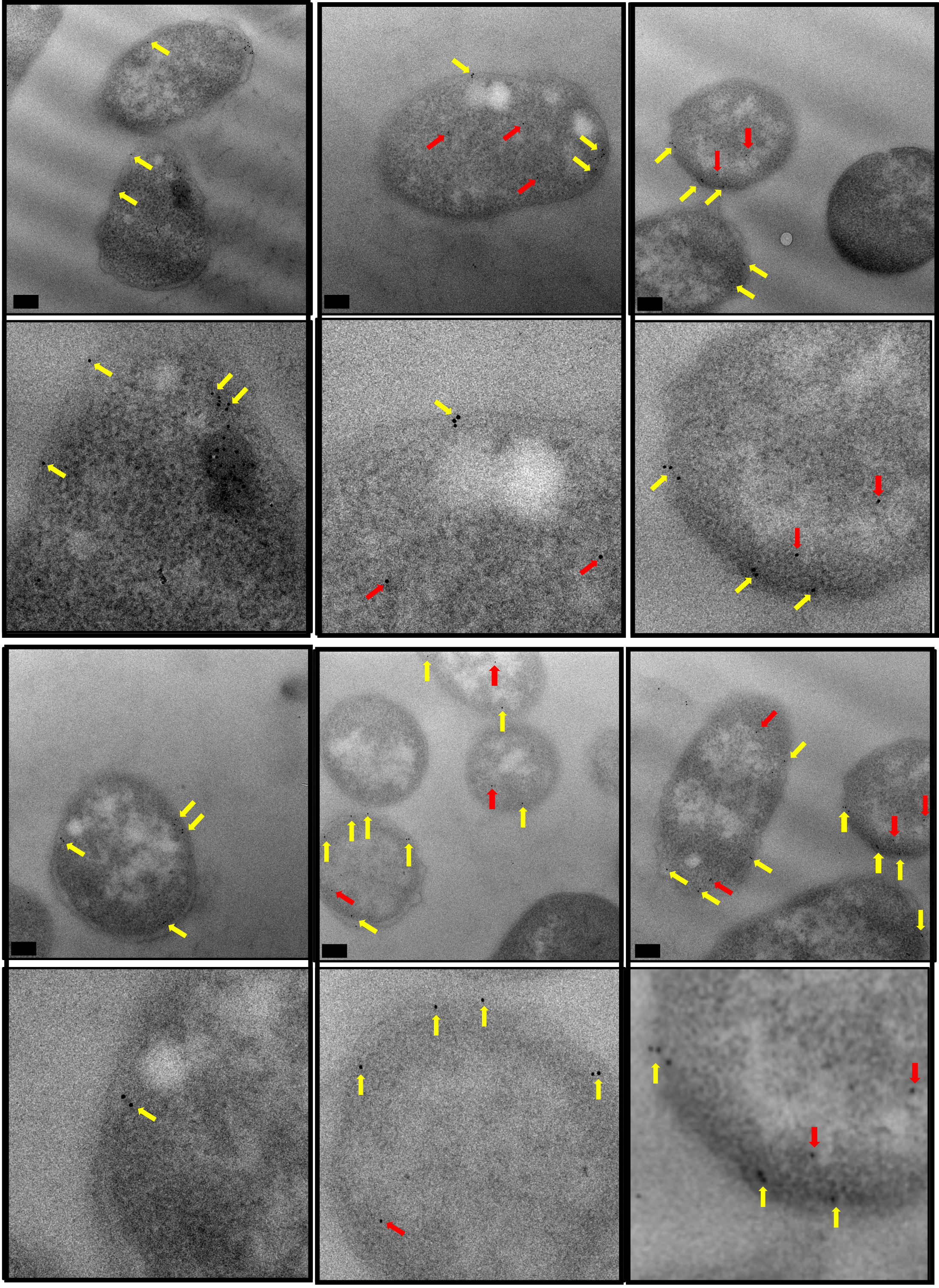

Figure S3. Electron micrographs of *E. coli* cells, overexpressing TorA-hGH (WT precursor), immunogold-labelled following primary antibody detection against hGH protein. Ultrathin sections of *E. coli* cells overexpressing TorA-hGH (WT precursor) were immunolabelled using a polyclonal antibody raised against hGH (shown in rows 1 and 3, with rows 2 and 4 showing close-ups of individual gold particles from rows 1 and 3, respectively). At 1hr 45 min after induction, hGH was found to exhibit a random distribution in the inner membrane (yellow arrows) and was also present in the cytoplasm (red arrows). Images were taken on a JEOL 2010F at 15,000X magnification. Scale bar = 200 nm.
