# Supplementary material for: Characterization of a novel method for the production of single‐span membrane proteins in Escherichia coli
Source: Biotechnol Bioeng. 2019 Jan 19;116(4):722–33. doi: 10.1002/bit.26895 (PMC6492203; doi:10.1002/bit.26895)
Supplement: Supplementary file 4 — Supporting information [file BIT-116-722-s004.pdf]

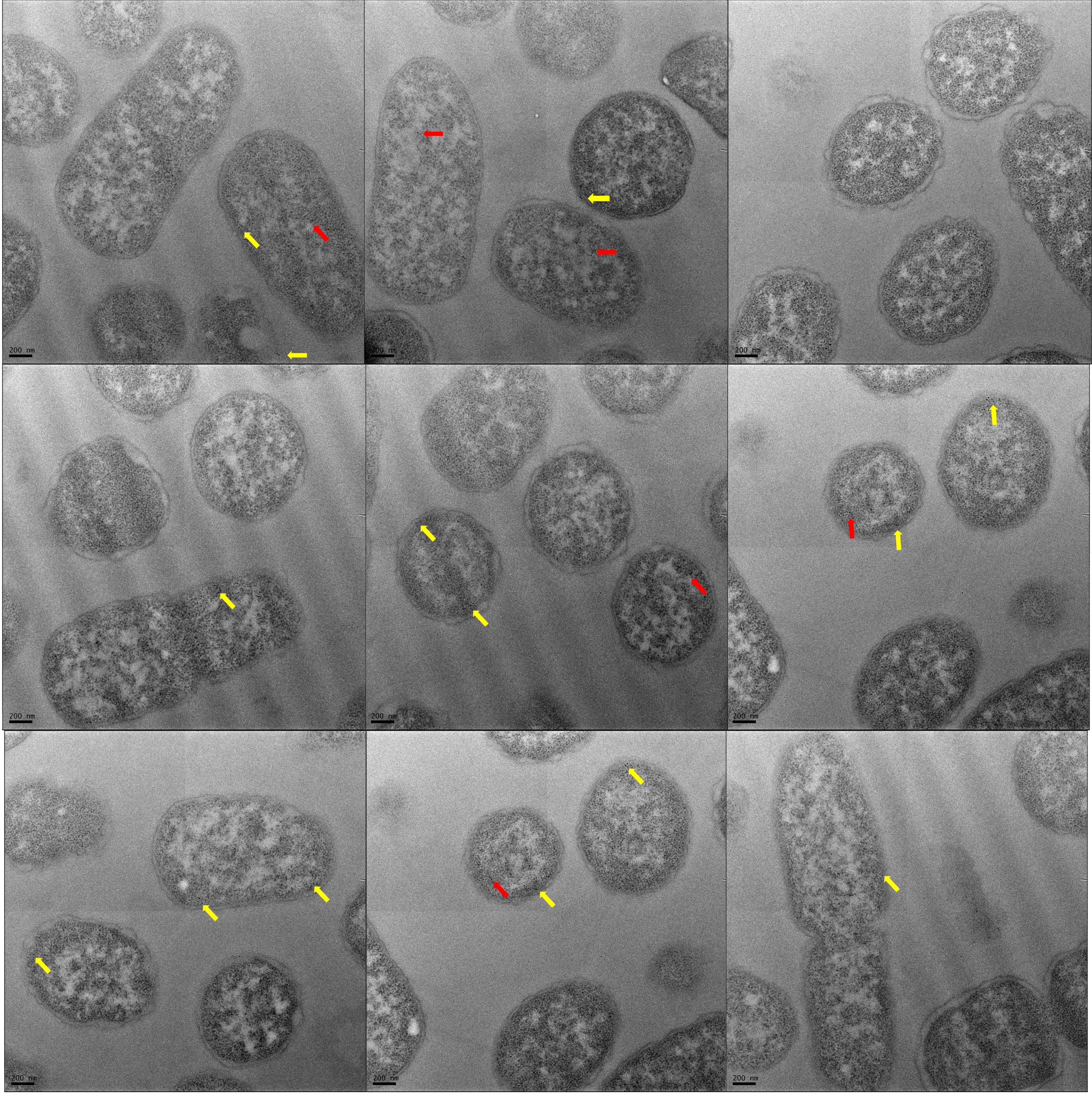

Figure S4. Electron micrographs of *E. coli* cells, lacking expression of TorA-hGH, immunogold-labelled following primary antibody detection against hGH protein. Ultrathin sections of *E. coli* cells lacking expression of TorA-hGH were immunolabelled using a polyclonal antibody raised against hGH to serve as a negative control. An abundance of cells lacking gold binding were detected. A minority of cells bound gold at the cytoplasm (red arrows) and the inner membrane (yellow arrows). However, the difference between these control cells and cells overexpressing TatA protein was confirmed to be statistically significant. Images were taken on a JEOL 2010F at 15,000X magnification. Scale bar = 200 nm.
