# Supplementary material for: Characterization of a novel method for the production of single‐span membrane proteins in Escherichia coli
Source: Biotechnol Bioeng. 2019 Jan 19;116(4):722–33. doi: 10.1002/bit.26895 (PMC6492203; doi:10.1002/bit.26895)
Supplement: Supplementary file 5 — Supporting information [file BIT-116-722-s005.pdf]

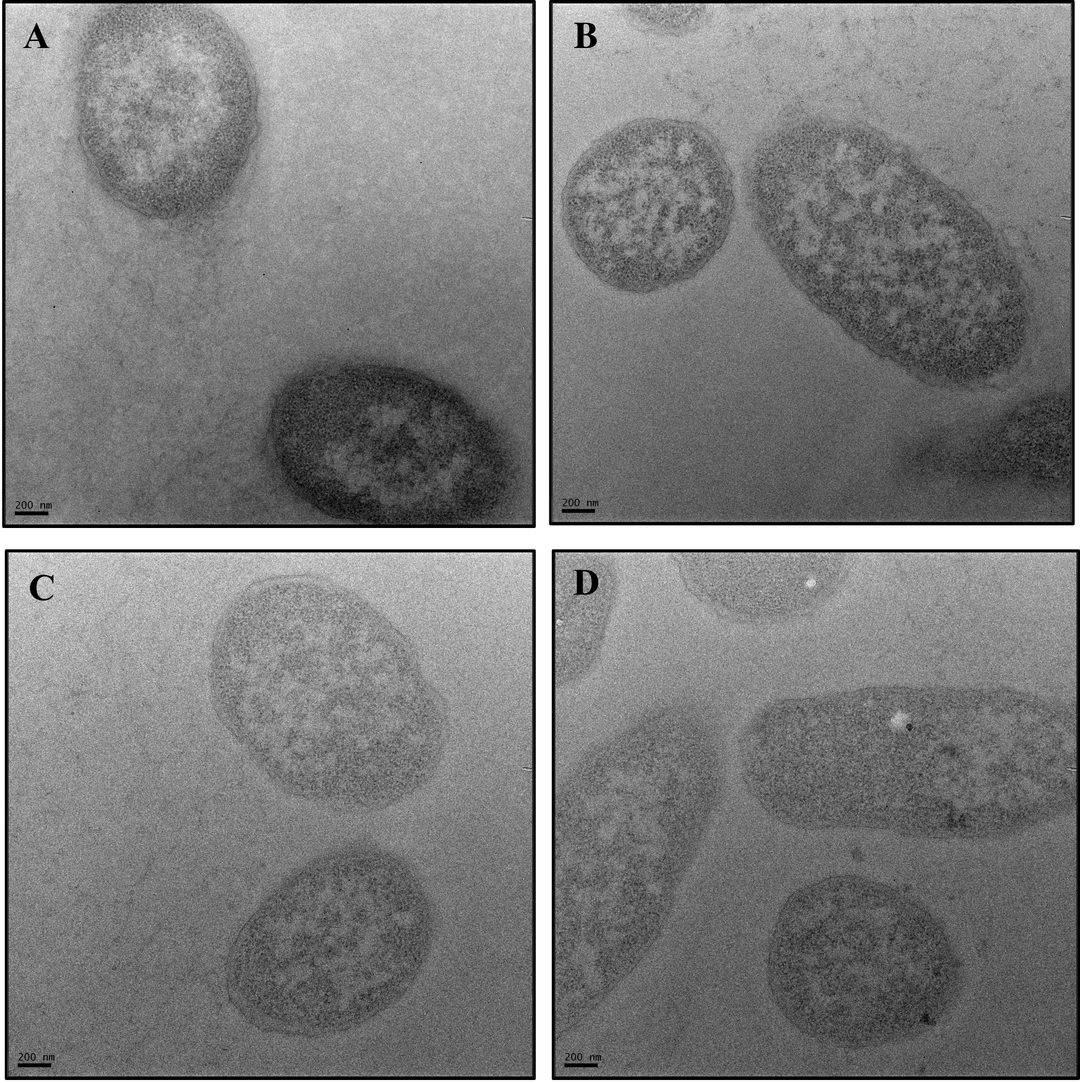

Figure S5. Electron micrographs of *E. coli* cells, overexpressing TorA-hGH, immunogold labelled omitting the use of primary antibody and using a different secondary antibody.

Ultrathin sections of *E. coli* cells overexpressing TorA-hGH (WT or mutant precursor- A and B, respectively), were immunolabelled without the use of a primary, polyclonal antibody raised against hGH and thus labelled with a gold-conjugated secondary antibody only. The lack of gold particles in both samples (A and B) confirmed that non-specific binding was attributable to solely the primary antibody. The same cell types (WT or mutant precursor- C and D, respectively), were immunolabelled with a gold-conjugated secondary antibody directed towards a different animal species. The lack of gold particles in both samples (C and D) confirmed that the cells do not have a non-specific attraction for gold particles. Images were taken on a JEOL 2010F at 15,000X magnification. Scale bar = 200 nm.
